# *In vivo* models to study gastrointestinal *Salmonella* infections

**DOI:** 10.1080/19490976.2026.2659456

**Published:** 2026-04-15

**Authors:** Ines Thiers, Bram Lories, Hans Steenackers

**Affiliations:** a Centre of Microbial and Plant Genetics (CMPG), Department of Microbial and Molecular Systems, KU Leuven, Leuven, Belgium

**Keywords:** Non-typhoidal *Salmonella*, model systems, gastrointestinal infection

## Abstract

Given that non-typhoidal *Salmonella* (NTS) remains a leading cause of foodborne infections worldwide, efforts to detect and combat these pathogens continue to be a major focus of research. Enhancing *Salmonella* prevention and treatment strategies requires a comprehensive understanding of infection dynamics and host‒pathogen interactions. Although *in vitro* models can provide preliminary insights into these interactions, animal studies remain crucial in grasping the complexities. In this regard, invertebrate systems can be valuable for conducting high-throughput screenings, while higher-order animals can offer insights into more complex interactions. As no model is capable of fully reflecting the natural infection conditions, a combinatory approach, integrating insights from different models, is often required. Here, we review the state-of-the-art *in vivo* models used to study NTS infections, including the *Caenorhabditis elegans*, *Danio rerio*, chicken, murine, bovine, and pig models. By balancing their employability with their physiological and ecological relevance, we provide a thorough comparison of these models.

## Introduction

The persistently high prevalence of non-typhoidal *Salmonella* (NTS) has generated and continues to generate considerable interest in understanding the pathogen's pathology and transmission. For this purpose, studies using both *in vitro* and *in vivo* models have proven valuable. *In vitro* models can provide useful preliminary insights, thereby adding to the replacement principle of the 3Rs (replacement, reduction, and refinement). The 3Rs form an important legal and practical framework to promote ethical and humane animal research.^1^^,^^2^ In the context of replacement, more advanced *in vitro* models, including the intestine chip and the simulator of the human intestinal microbiome ecosystem (SHIME), are being developed to better mimic physiological conditions.^3^^,^^4^ Still, no current model succeeds in combining key factors, including peristalsis, mucus production, innate and adaptive immune signaling, and the presence of a fully grown microbiome for longer than one week.^5^ As such, no *in vitro* model currently fully captures the *in vivo* complexity, necessitating translation of the findings to animal models. When moving towards *in vivo* studies, researchers must carefully balance the potential harm to animals against the scientific value of their research. This so-called “harm-benefit analysis” involves minimizing animal harm by implementing the 3R principles, as well as ensuring scientific reproducibility and generalizability.^1^^,^^2^^,^^6^ Integrating these principles allows for research that is both ethically responsible and scientifically justifiable.

Depending on the research question, an appropriate *in vivo* model must mimic relevant infection conditions while balancing reproducibility, ease of handling, cost, and throughput. Several review papers have previously provided valuable and comprehensive overviews of the experimental systems available to study *Salmonella* infections.^7-9^ Depending on their scope, these works focused on comparisons between enteritis and typhoid models, or on systems relevant to vaccine development. Complementing these prior assessments, a review dedicated to specifically evaluating all non-typhoidal models would be useful. As comparatively less attention has been given to physiological and anatomical differences among hosts before, the present review concentrates on anatomical and physiological features that may influence NTS infection outcomes. Some models involve natural hosts, offering insights into the pathogen’s transmission and persistence in the environment, thereby helping to understand foodborne outbreaks and their spread in agricultural settings. Other models rely on typical laboratory models and aim to draw parallels to human disease and pathogenesis. Table 1 summarizes the properties of all discussed models. Before going over the different model systems, we provide a brief overview of human physiology, anatomy, and infection, to allow comparison.

**Table 1.** Overview of the discussed models.

MODEL	Immune response	Intestinal anatomy	*Salmonella* infection	Advantages/disadvantages
*Caenorhabditis elegans*	– Simple innate immune system (e.g. no specialized phagocytotic cells)^10^ – Conserved signaling pathways present such as MAPK, TGF-*β*, DAF/IGF and UPR^10-17^ – Detect DAMPs^18^^,^^19^ – No adaptive immunity	– Simple gastrointestinal system (e.g. no different intestinal segments)^20^ – Intestinal microbiome dominated by Proteobacteria^21-23^	– Not a natural host – No invasion of epithelial cells^24^ – Induction of MAPK signaling and production of antimicrobial components (e.g. lysozymes and saposin-like proteins)^25^^,^^26^ – Authophagy and PCD protect against *Salmonella*^27^^,^^28^ – SPI-1 and SPI-2 interact with immune pathways^26^^,^^29^	– Highly simplified compared to humans (−) – Easy to maintain and manipulate (+) – Cheap, rapid and high throughput analysis (+) – Transparent body allows life imaging (+) – Wide set of tools available (+)
*Danio rerio*	– Innate immune system with leukocytes and granulocytes^30^^,^^31^ – Simple adaptive immune system (e.g. lacks PPs and lymph nodes, and has only three Ig classes)^32-34^ – Detect PAMPs via PRRs^35^^,^^36^ – Temporal separation innate & adaptive immune development^31^^,^^32^^,^^37^^,^^38^	– Simple gastrointestinal system (e.g. lacks a true stomach)^31^^,^^37^ – Intestinal microbiome dominated by Proteobacteria^39-41^	– Not a natural host – Predominant colonization in cloaca and mid-and posterior gut^42^ – Epithelial invasion can occur^43^^,^^44^ – Induction of MAPK & MyD88 pathways, and expression of cytokines leading to inflammation^45^^,^^46^ – Neutrophil recruitment to clear infection^43^^,^^44^ – Virulence plasmid and SPI-1 important for inflammation^47-49^	– Highly simplified compared to humans (−) – Easy to maintain and manipulate (+) – Cheap, rapid and high throughput analysis (+) – Transparent embryos allow life imaging (+) – Wide set of tools available (+)
Chicken model	– Innate immune system with leukocytes and granulocytes (but heterophils instead of neutrophils)^50^ – Adaptive immune system with three Ig classes^51^^,^^52^ – Detect PAMPs via PRRs^53^ – Unique primary (Bursa of Fabricus) and secondary (cecal tonsils and Meckel’s diverticulum) lymphoid organs^53^^,^^54^	– Different organization of gastrointestinal system compared to humans (e.g. has a crop, two-section stomach, two ceca)^55-57^ – Intestinal microbiome composition similar to humans at phylum level (dominated by Firmicutes and Bacteroidetes)^58-60^ – Microbiome most dense and diverse in cecum^59^	– Natural host for NTS – Depending on chicken strain, different sensitivity to NTS – Predominant colonization in cecum^61^ – Invasion of epithelial and M cells^62^ – Induction of cytokines, leading to inflammation^63^^,^^64^ – Heterophil recruitment to site of infection^63^^,^^64^ – Role of SPI-1 and SPI-2 ambiguous^65-69^	– Different physiology than humans (−) – Ecological relevance (+) – Wide set of tools available (+) – Expensive (−) – Relatively low throughput (−)
Murine colitis model	– Innate immune system with leukocytes and granulocytes similar to humans (with differences in e.g. signaling pathways and abundances)^70^^,^^71^ – Adaptive immune system similar to humans^70^^,^^71^ – Detect PAMPs via PRRs	– Gastrointestinal system similar to humans, though less than for pigs (e.g. has a two-chamber stomach and a longer small intestine)^72^^,^^73^ – Intestinal microbiome composition similar to humans at phylum level (dominated by Firmicutes and Bacteroidetes)^74-76^ – Microbiome most dense and diverse in cecum and colon^77^	– Depending on mice strain, different sensitivity to NTS^78-81^ – Predominant colonization in cecum^82^^,^^83^ – Invasion of epithelial and M cells^84^ – Induction of cytokines, leading to inflammation^82^^,^^83^ – Recruitment of neutrophils to site of infection^84^^,^^85^ – SPI-1 important for colonization and SPI-2 for intracellular survival^86^	– Different infection models: Streptomycin-pretreated model,^84^^,^^87^ gnotobiotic mice,^88^^,^^89^ germ-free mice^90^… (+) – Comparable physiology to humans (+) – Relatively easy to maintain and manipulate (+) – Wide set of tools available (+) – Relatively low throughput (−)
Bovine model	– Innate immune system with leukocytes and granulocytes similar to humans^91^ – Adaptive immune system similar to humans^91^ – Detect PAMPs via PRRs	– Different organization gastrointestinal system compared to humans (e.g. a four-chamber stomach, a larger small and large intestine, lack of an appendix)^92^ – Intestinal microbiome composition similar to humans at phylum level (dominated by Firmicutes and Bacteroidetes)^93^^,^^94^ – Microbiome most dense and diverse in reticulorumen^93^^,^^94^	– Natural host – Predominant colonization in ileum^95-97^ – Invasion of epithelial and M cells ^95-97^ – Recruitment of neutrophils to site of infection^96^^,^^98^^,^^99^ – SPI-1 and SPI-2 important for enteric and systemic infection^100^^,^^101^	– Different infection models: ileal loop model^102^ or infection in calves^103^ – Mimics human infection well (+) – Ecological relevance (+) – Expensive (−) – Fewer tools available (−) – Moderate throughput for ileal loop model (+), no high throughput for cattle infection model (−) – Logistic challenges (−)
Pig model	– Innate immune system with leukocytes and granulocytes similar to humans^104^^,^^105^ – Adaptive immune system similar to humans^104^^,^^105^ – Detect PAMPs via PRRs	– Gastrointestinal system similar to humans (with small differences e.g. larger cecum, lack of an appendix)^106-108^ – Intestinal microbiome composition similar to humans at phylum level (dominated by Firmicutes and Bacteroidetes)^109-112^ – Microbiome most dense and diverse in large intestine^113^	– Natural host – Predominant colonization in ileum^114-116^ – Invasion of epithelial and M cells^117^ – Induction of cytokines, leading to inflammation – Recruitment of neutrophils to site of infection^117^ – SPI-1 important for intestinal colonization, but not for palatine tonsil colonization^118-120^ – SPI-2 important for systemic colonization^121^	– Different infection models: ileal loop model^122^^,^^123^ or infection in pigs^124^ – Mimics human infection well (+) – Ecological relevance (+) – Wide set of tools available (+) – Expensive (−) – Moderate throughput for ileal loop model (+), no high throughput for pig infection model (−) – Logistic challenges (−)

## Humans

### Immunity

Humans, like other vertebrates, possess both innate and adaptive immune systems that function in a coordinated manner to protect against infection. The innate immune system provides the first line of defence, relying on a diverse set of cellular components.^125^ Dendritic cells, for instance, act as key sentinels, continuously surveying tissues and presenting antigens to initiate downstream immune responses. Next, granulocytes protect against infection, with neutrophils releasing antimicrobial enzymes such as *α*-defensins, and reactive oxygen species (ROS).^126^^,^^127^ Macrophages in turn support tissue homeostasis, perform phagocytosis, and produce antimicrobial molecules, including nitric oxide. Lastly, natural killer (NK) cells and innate-like lymphocytes provide rapid cytotoxic activity and help shape early immune responses.^128^ The humoral arm of innate immunity comprises the complement system, which promotes pathogen lysis, opsonization, and clearance, as well as soluble mediators such as cytokines and chemokines that coordinate intercellular communication.^125^ Here, the multiprotein complex inflammasome serves as an important sensor, releasing pro-inflammatory cytokines to induce pyroptosis.^129^

Pathogen recognition is typically mediated by pathogen recognition receptors (PRRs), which detect pathogen associated molecular patterns (PAMPs), such as lipopolysaccharide (LPS) and peptidoglycan. Humans encode five types of PRRs, of which the Toll-like receptors (TLRs) are the best described.^127^^,^^128^ Upon TLR activation, downstream signaling cascades such as MyD88 are activated, eventually resulting in cytokine production.^130^

The adaptive immune system is mediated by B and T lymphocytes. In humans, T lymphocytes or T cells originate in the bone marrow and undergo maturation in the thymus, whereas B lymphocytes or B cells develop and mature in the bone marrow before migrating to peripheral lymphoid organs such as the spleen and lymph nodes, which are present across the body. The human adaptive immune system produces five immunoglobulin (Ig) classes: IgA, IgM, IgG, IgD, and IgE.^127^^,^^128^ Secretory IgA plays a central role in mucosal immunity, particularly in the gastrointestinal tract.^131^

The intestine exhibits several specialized immune features that reflect its constant exposure to dietary antigens and the microbiota. Paneth cells, for instance, located at the base of the crypts in the small intestine, secrete antimicrobial peptides, including *α*-defensins and lysozymes, to promote microbial control and epithelial barrier maintenance.^132^ The gut also contains organized lymphoid structures collectively referred to as gut-associated lymphoid tissue, comprising both isolated lymphoid follicles and aggregated follicles known as Peyer’s patches (PPs). PPs are covered by follicle-associated epithelium containing specialized microfold (M) cells. These M cells lack a dense glycocalyx, facilitating the transcytosis of luminal antigens and microorganisms to underlying immune cells such as dendritic cells, B cells, and T cells.^133^ Finally, epithelial cells themselves can also produce antimicrobial compounds, such as *β*-defensins and cathelicidins, offering protection against invaders.^134^^,^^135^

### Gastrointestinal tract

Like all mammals, the human gastrointestinal tract (GIT) consists of an esophagus, a stomach, a small intestine – subdivided into the duodenum, jejunum, and ileum – and a large intestine, which includes the cecum (in some mammals), colon (ascending, transverse, descending, and sigmoid segments), and rectum. Each compartment is composed of four primary tissue layers: mucosa, submucosa, muscularis externa, and serosa. The mucosa itself consists of three sublayers: an epithelial layer, the lamina propria, and the muscularis mucosae.^136^^,^^137^ In the small intestine, the mucosal surface is folded into circular folds and further bent by villi and microvilli, dramatically increasing the surface area for nutrient absorption. The apical surfaces of the microvilli are covered by a glycocalyx, which contains digestive enzymes for nutrient processing.^138^ In the large intestine, such villi and microvilli are absent, but intestinal crypts are abundant.

Although the general organization of the GIT is conserved across mammals, anatomical difference exist based on diet preferences. As omnivores, humans possess a single-chambered, glandular stomach, rely primarily on autoenzymatic digestion, and use the colon as the main site of microbial fermentation.^139^ The human cecum is relatively small and gives rise to the vermiform appendix, which is proposed to serve immunological functions and act as a microbial reservoir.^140^

At birth, the human gut microbiome is premature, with the first colonizers depending on maternal factors and the mode of delivery. During the first month of life, the phyla Actinobacteria and Proteobacteria typically dominate the gut. Over the subsequent 2–3 years, the microbiome transitions towards an adult-like configuration, characterized by increased abundance of Firmicutes and Bacteroidetes. By early childhood, the microbial community stabilizes, although it remains dynamic throughout life.^141^

As in most mammals, the microbiome shifts throughout the human GIT. The bacterial density and diversity increase in the distal direction, with the colon harboring the highest microbial load. The small intestine is generally enriched in rapidly growing facultative anaerobes, while the large intestine is dominated by obligate anaerobic, short-chain fatty acid (SCFA)-producing species.^142^ The microbial composition in humans is further shaped by host genetics, diet, lifestyle, geography, antibiotic use, and disease status, resulting in great inter-person variability.

### *Salmonella* infection

Humans infected with NTS typically present acute gastroenteritis characterized by diarrhea, fever, and abdominal cramps. Nausea, vomiting, and headaches may also occur, but are less frequent.^143^ Endoscopic and histopathological analyses of infected patients have demonstrated mucosal edema, disruption of the mucus layer, epithelial damage, and prominent polymorphonuclear neutrophil infiltration of the rectal and colonic mucosa.^144^ In immunocompetent individuals, the infection is typically self-limiting, with neutrophil recruitment contributing to pathogenic clearance.^43^

Despite these clinical and histological observations, there is little mechanistic understanding of the infection process in humans specifically. The current mechanistic models stem from *in vitro* and animal studies, which, as explained below, do not fully imitate human infection. Direct validation of this integrative model in humans remains challenging, as controlled human infection studies with NTS are rare. Recently, a controlled human infection model has been established at Imperial College London.^145^ This platform has the potential to provide more direct insight into host–pathogen interactions in humans and may help bridge the gap between experimental models and naturally occurring clinical disease.

## Invertebrate model systems

Invertebrate model organisms offer advantages, including ease of manipulation, high-throughput screening, and low costs.^7^ Although their immunological and gastrointestinal systems are highly simplified compared to vertebrates, they can be useful to obtain preliminary insights. For instance, since these organisms naturally inhabit microbe-rich environments, they have evolved interesting microbe‒host interactions and defense strategies. Despite lacking adaptive immunity, invertebrates possess innate immune signaling pathways that share similarities with those observed in vertebrates.^146^ Therefore, invertebrate models can serve as a bridge, balancing the simplicity and scalability of less relevant *in vitro* models with the complexity and relevance of *in vivo* models. Still, results obtained from invertebrate models cannot be directly translated to vertebrates, requiring confirmation in higher-order models.

Initially, these animals were thought not to feel pain or suffer, thereby partially fulfilling the replacement principle of the 3Rs. However, this point of view has been widely debated over the last few years.^147^^,^^148^ In this review, we will particularly focus on the nematode model to study NTS. While *Drosophila melanogaster* and *Galleria mellonella* also represent commonly used invertebrate models with many practical advantages, they are limited to studying systemic *Salmonella* infections, as the pathogen is typically introduced via injection into the hemocoel.^149-153^ As such, these models will not be discussed here.

### Caenorhabditis elegans

Over the past half-century, *C. elegans* has emerged as one of the most widely recognized and favored invertebrate model organisms. This free-living roundworm normally inhabits decomposing plant matter and can easily travel between locations using vectors such as snails and isopods. Its small size, transparent body, rapid life cycle, and ease of manipulation make it an attractive laboratory model. Additionally, *C. elegans* mainly emerges as a hermaphrodite, yielding approximately 300 identical progeny per animal, thus facilitating high-throughput screening and large-scale experiments.^18^^,^^24^^,^^154^

Originally, *C. elegans* was employed in the field of genetics and developmental biology. However, over the last 15 y, *C. elegans* has also appeared to be a suitable model in the field of immunity and infectious diseases, with several studies focusing on NTS.^7^^,^^24^^,^^25^^,^^28^^,^^155-160^

#### Immunity

Given its bacterivorous nature, *C. elegans* naturally hosts a wide number of pathogenic and symbiotic bacteria. Therefore, it has developed complex mechanisms to differentiate beneficial from harmful microorganisms. To protect itself from pathogens, it employs a wide array of defense strategies, including innate and learned aversion,^161-163^ a pharyngeal grinder,^164^ a protective exoskeleton,^165^ and, interestingly, an inducible innate immune system. This innate immune system precedes the one in higher-order organisms.^18^^,^^166^ Although the complexity of immune signaling is consequently highly reduced, ancestral signaling networks, including the MAPK, transforming growth factor beta (TGF-*β*), the DAF/insulin-like growth factor (IGF), and the unfolded protein response (UPR) pathways are present.^10-17^ These pathways lead to cytokine induction in mammals, which activate antimicrobial compounds such as lysozymes, saposin-like proteins, antibacterial factors, and ROS, in nematodes.^18^^,^^25^ Notably, worms lack MyD88 and ReL/NF-κβ homologs.^161^^,^^167^ Also, specialized phagocytic cells are absent, though neighboring cells can perform phagocytic activities to remove dying cells using the CED pathways.^10^^,^^168^^,^^169^

Worms contain a TLR homolog, namely, TOL-1. Unlike in mammals, where TLRs play a significant role in pathogen recognition and induction of inflammation, TOL-1 mainly contributes to avoidance behavior in response to pathogens.^161^^,^^167^ In fact, worms do not recognize PAMPs, but rather react upon damage-associated molecular patterns (DAMPs). Examples include intestinal distention due to bacterial colonization and pathogen-induced mitochondrial damage.^18^^,^^19^ Worm’s C-type lectin domain-containing proteins do recognize and bind bacterial cell walls, but their role in immune signaling is ambiguous.^170^

While worms do not possess an adaptive immune system, they exhibit a form of immune memory through immune priming. Here, exposure to pathogenic or non-pathogenic bacteria induces epigenetic changes, resulting in a primed state where immune components are maintained above baseline levels.^171-173^ As worms rely on DAMPs, non-bacterial stressors, such as elevated temperatures, can also trigger a primed immune state by activating the innate immune signaling pathways. This should be taken into account when using the nematodes as models for infectious diseases.

#### Gastrointestinal tract

**Figure 1.**
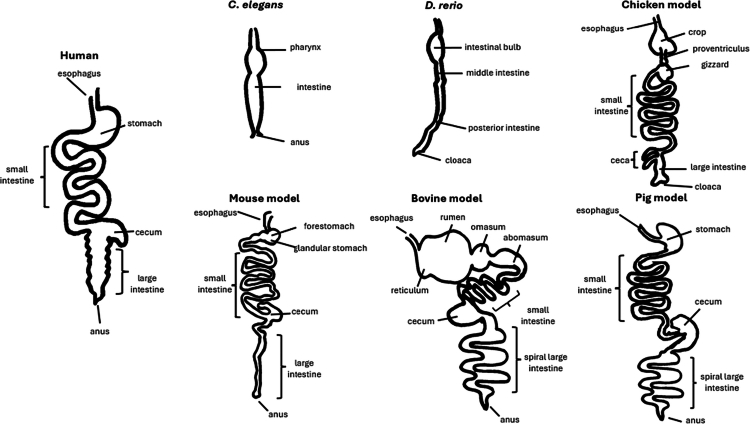
Schematic overview of the gastrointestinal tract of the different model systems.^176-180^

*C. elegans’* digestive tract consists of a buccal cavity, a pharynx, an intestine, and an anus, as schematized in Figure 1.^51^ The pharynx, which functions similarly to the esophagus, actively pumps food into the intestine.^174^ The pharynx and intestine are separated by a grinder, which mechanically and enzymatically breaks down microbes.^164^ The partially digested material then moves to the intestine, where additional enzymatic processing occurs. The intestine itself comprises 20 cells arranged in a tubular structure lined with microvilli and a glycocalyx on the apical side facing the lumen.^175^ Interestingly, somatic cells in *C. elegans* cannot regenerate, leaving the intestinal cells unchanged throughout the organism’s lifespan.^20^

In its natural environment, *C. elegans* hosts a rich and diverse microbiome that fulfills functions similar to the mammalian microbiome, such as enhancing immunity, promoting colonization resistance, and providing health benefits. The worm’s microbiome is dominated by members of the Proteobacteria, Bacteroidetes, Firmicutes, and Actinobacteria phyla.^21-23^ Healthy worms typically harbor a higher proportion of *α*-Proteobacteria, whereas diseased nematodes exhibit an increased abundance of Bacteroidetes. Interestingly, much of this microbial composition appeared reproducible on various substrates, indicating the presence of a worm-specific microbiome that is relatively independent of its environmental niche.^23^

In laboratory settings, *C. elegans* microbiota can be completely removed through bleaching, allowing for precise control of microbial colonization. Most commonly, the worms are cultured on *Escherichia coli* OP50 as a food source.^181^^,^^182^ While *E. coli* OP50 typically does not colonize the worm's intestine effectively, as the worm age and their immune system becomes less efficient, it can persist and accumulate in the gut.^159^

#### *Salmonella* infection

NTS serovars do not naturally infect nematodes, but infection can be simulated in the lab by replacing the feed of *C. elegans* by the serovar of interest. Upon ingestion, the pathogen gradually destroys the pharyngeal grinder and, in turn, colonizes the intestinal lumen of the worm. This causes persistent infection, significantly reducing the nematode lifespan.^24^^,^^183^ Unlike in humans, no epithelial invasion takes place.^24^ The worm model thus mainly enables investigation of host–pathogen interactions occurring during intestinal persistence, such as epithelial stress responses, and conserved innate immune signaling pathways. For instance, as a response to the presence of *Salmonella,* the nematode induces the MAPK and DAF/IGF signaling pathways, resulting in the production of antimicrobial components such as lysozymes and saposin-like proteins.^25^^,^^26^

As mentioned above, *Salmonella* is incapable of invading epithelial cells in the worm, questioning the role of SPI-1 and SPI-2 effectors in this model.^24^ Still, life span analyses showed that deletion of SPI-1 and SPI-2 increased worm survival and lowered immune activation, suggesting a role for these effectors during the infection process.^159^^,^^160^ Subsequent studies showed that the SPI-1 effector SptP interferes with MAPK signaling, and that other SPI-1 and SPI-2 effectors provide resistance to the host’s antimicrobial peptides.^26^^,^^29^ The SPI-1 and SPI-2 effectors thus seem to interact with immune signaling pathways, thereby affecting virulence. Interestingly, similar effects of SptP on the MAPK pathway in cell line experiments have been observed, suggesting that some innate immune responses are highly conserved.^184^ The worm model can thus be useful to identify conserved signaling molecules controlling intestinal infection.

When intestinal-expressed autophagy genes are inhibited, *Salmonella* do not remain extracellular, but can invade the epithelial lining.^27^ This finding suggests a critical role for intestinal autophagy in offering protection, corresponding to findings from cell line studies.^185^ Worms can thus be useful to characterize fundamental principles of immunity. Also programmed cell death appears important, as cell death mutants showed hyper susceptibility to *Salmonella* infection.^28^

The use of *C. elegans* offers several advantages.^18^^,^^21^^,^^186-188^ First, the invertebrate allows simple, high-throughput, and cost-effective maintenance and manipulation.^18^^,^^21^^,^^186-188^ Second, the nematode’s transparent body enables straightforward visualization of *Salmonella* within the intestinal lumen.^182^ Additionally, feeding *C. elegans* a controlled bacterial diet or using specific mutant strains facilitates the study of *Salmonella*–microbiome and *Salmonella*–host interactions, respectively.^21^^,^^189^^,^^190^ Still, the worm remains a highly simplified system, lacking key immune and GIT components and infection dynamics as observed in mammals. This complicates direct translation of findings to ecologically and societally relevant settings.

## Vertebrate model systems

While invertebrate models offer primary insights into *Salmonella*–host interactions, their lack of an adaptive immune system and the simple structure of their GIT necessitate further validation in the physiologically more relevant vertebrate models.

Increased relevance comes with greater complexity. All vertebrates possess both innate and adaptive immune systems, with the anatomy, maturation, and signaling mechanisms varying across species.^191^ Likewise, the GIT of vertebrates, while fundamentally similar in design, can differ in size and function depending on the dietary requirements, as schematized in Figure 1.^192^ Consequently, the infection profile of *Salmonella* can vary between different models. In the following sections, the use of zebrafish, mice, chicken, cattle, and pigs for studying gastrointestinal *Salmonella* infections will be discussed.

Note that research involving higher-order animal models requires prior ethical approval to ensure animal welfare and scientific justification. These concerns include, but are not limited to, minimizing pain and distress, optimizing the sample size to achieve statistically relevant results, and ensuring scientific validity. Protocols must be reviewed and approved by appropriate institutional committees to assess compliance with ethical and regulatory standards. This oversight is essential for responsible and credible research.

### Danio rerio

The zebrafish, a freshwater species native to South Asia, is a widely used model organism in biomedical research. It combines the advantages of invertebrate models, such as small size, low cost, short generation time, and suitability for large-scale screening, with the anatomical and physiological complexity of vertebrates. Moreover, its *ex utero* development and transparent embryos allow for real-time, in situ observation of various developmental processes, immune activation, and pathogen‒host interactions.^37^^,^^39^^,^^193^ As a result, a number of research groups have utilized zebrafish to study *Salmonella* infections.^43^^,^^44^^,^^47^^,^^48^^,^^194^^,^^195^

#### Immune system

Despite being a lower vertebrate, zebrafish contain an adaptive and innate immune system that possess features similar to the mammalian one. For instance, the innate immune system comprises leukocytes, including macrophages, and granulocytes, such as neutrophils, which serve as the first line of defense against pathogens.^30^^,^^31^ The innate immune response also largely relies on PRRs such as TLRs. Zebrafish exhibit a broad repertoire of TLRs with over 20 variants, approximately half of which have human orthologs.^35^^,^^36^ As in mammals, PRR activation typically triggers cytokine signaling and the subsequent secretion of antimicrobial molecules. An example of this is the presence of two IL-8 homologs in zebrafish, Cxcl8-I1 and Cxcl8-I2, which play key roles in fighting *Salmonella.*^194^^,^^196^ Furthermore, zebrafish encode inflammasome homologs, similarly inducing pyroptosis upon pathogen recognition.^197^

The zebrafish adaptive immune system has T and B lymphocytes mounting antigen-specific immune responses. Unlike in humans, B cell development occurs in the kidney marrow, which serves as the primary lymphoid organ, together with the thymus. The main secondary lymphoid organ in zebrafish is the spleen, as they lack lymph nodes and PPs.^32^^,^^33^ Furthermore, zebrafish encode only three (Ig) classes, namely, IgD, IgM and IgZ, instead of five in humans.^34^

Interestingly, the development of the innate and adaptive immune systems follows a distinct timeline in zebrafish. While innate immunity is present from the beginning of embryogenesis, the adaptive immune response begins to mature only around 4 weeks after fertilization.^31^^,^^32^^,^^37^^,^^38^ This temporal separation provides a unique opportunity to separately study innate and adaptive immune responses upon infection.

Importantly, the zebrafish’ immune system is highly sensitive towards environmental stress. Therefore, to ensure reproducible and reliable results, it is crucial to maintain a stress-free environment with consistent feeding and monitoring.^198^

#### Gastrointestinal tract

In terms of development, biological function, and overall organization, the zebrafish GIT broadly resembles that of mammals. Functional diversification along the anterior‒posterior axis is preserved, with the anterior segment being primarily responsible for lipid and protein absorption, while the posterior segment plays a key role in water and ion absorption. Additionally, both zebrafish and mammalian GITs contain enterocytes, enteroendocrine cells, and goblet cells, which perform conserved functions and secrete functionally equivalent enzymes.^31^^,^^37^^,^^38^

However, there are important architectural differences. Unlike the mammalian GIT, which consists of four distinct segments – the esophagus, stomach, small intestine, and large intestine – the zebrafish GIT comprises only three segments: the anterior intestinal bulb, middle intestine, and posterior intestine. The zebrafish thus lacks a true stomach. While the anterior intestinal bulb has a slightly larger lumen than the rest of the intestine and has been suggested to function as a reservoir similar to a stomach, it does not undergo acidification and thus does not fully mimic the stomach’s function.^31^^,^^37^ In fact, the intestinal bulb is functionally more analogous to the human duodenum. The middle intestine is in turn considered equivalent to the jejunum and ileum, while the posterior intestine better corresponds to the colon.^176^ The posterior segment terminates at the cloaca, which serves a shared outlet for both the gastrointestinal and urinary tracts.

Further structural distinctions include the absence of a submucosa and muscularis mucosa in the zebrafish gastrointestinal wall.^199^ Additionally, instead of forming villi, the intestinal tissue folds irregularly to increase the surface area.^200^ Furthermore, zebrafish lack specialized immune structures such as PPs and Paneth cells.^32^^,^^33^ Beyond these anatomical differences, zebrafish and mammals also diverge in their diets, body size, metabolic demands, and environmental conditions.^38^

These environmental conditions and diets also serve as major drivers shaping the gut microbiome. Unlike mammals, the zebrafish’ microflora is dominated by Proteobacteria, followed by Firmicutes, Actinobacteria and Bacteroidetes.^39-41^ The diversity and complexity highly varies across developmental stages: in early life, *α*-Proteobacteria are more prevalent, with a subsequent increase in *β*-Proteobacteria at the juvenile stage, followed by an expansion of Firmicutes in later stages.^38^ While the adult zebrafish microbiome is considered to be relatively stable, it remains influenced by both intrinsic and extrinsic factors, including gastrointestinal infections.

#### *Salmonella* infection

Zebrafish can be employed to study NTS infection by infecting the fish via immersion or microgavage.^201-203^ While immersion is more convenient and high throughput, it raises concerns about precise dosing.^204^ In contrast, microgavage is less straightforward, requiring specialized equipment and technical expertise, but it ensures greater accuracy.^205^ Both larvae and adult zebrafish have been used as infection models, leading to studies focusing on innate and adaptive immunity, respectively.

Following oral infection, *Salmonella* colonizes the zebrafish GIT, triggering inflammation.^44^^,^^206^ The MAPK and MyD88 pathways are typically upregulated, resulting in the induction of cytokines such as Cxcl-8, Cxcl-C1c, and IL-1β. Also, the complement system and apoptotic and proteolytic pathways are induced, further contributing to an inflammatory environment.^45^^,^^46^ Colonization and inflammation predominantly occur in the cloaca, and the mid- and posterior gut.^42^ While the majority of the bacterial cells reside in the mucosae, epithelial invasion can occur. The induced inflammation eventually results in neutrophil recruitment to clear the infection.^43^^,^^44^

Expression of the *Salmonella* plasmid virulence operon appears important for infection establishment in zebrafish, with the virulence gene *spvB* contributing to an inflammatory environment.^47^^,^^48^ SPI-1 effectors also induce an inflammatory response by promoting inflammasome activation and subsequent pyroptotic activities. Accordingly, zebrafish infected with SPI-1/SPI-2 deficient mutants exhibit increased survival and reduced inflammatory responses.^49^

The zebrafish serves as a valuable model for NTS studies, providing preliminary insights into host‒pathogen interactions with high-throughput capabilities. The temporal separation of innate and adaptive immunity, larval transparency, and the availability of transgenic, mutagenic, and sequencing tools enable efficient and straightforward experimentation.^37^^,^^39^^,^^193^ Nevertheless, similar to *C. elegans*, the relative simplicity of this model and the structural divergence of its immune and gastrointestinal systems from those of mammals restrict direct extrapolation, requiring confirmation in higher-order animal models.

### Chicken model

Poultry serve as the primary reservoir for NTS serotypes, facilitating transmission to humans through contaminated eggs or meat.^207^^,^^208^ Consequently, extensive research has been conducted on NTS infections in chickens. However, owing to substantial anatomical differences between birds and mammals, caution must be taken when extrapolating these findings to human infections.

#### Immunity

Broadly speaking, the avian immune system mirrors that of mammals, encompassing both innate and adaptive components, along with comparable organ structures and signaling pathways. However, the mechanisms underlying this organization and signaling often differ between the two groups. For instance, B cell maturation in birds does not occur in the bone marrow, but in the Bursa of Fabricius. The Bursa is a unique organ of the avian immune system, located as a diverticulum of the cloaca. Additionally, birds possess several distinct secondary lymphoid organs, such as the cecal tonsils and Meckel’s diverticulum, which are absent in mammals. Notably, avians lack lymph nodes, with the cecal tonsils likely fulfilling a similar function.^53^^,^^54^

Other key differences include variations in Ig classes and the diversification of the antibody repertoire. Specifically, chickens contain only three Ig isotypes, namely, IgA, IgY, and IgM, and thus lack orthologs of IgD and IgE. IgY is the major isotype transferred via the egg yolk and plays an important role in protection against pathogens.^51^^,^^52^

At the level of immune signaling, birds lack neutrophils and instead rely on heterophils.^50^ Despite this difference, birds generally possess most immunological families found in mammals, albeit with fewer and/or other members. Exceptions to this are the leukocyte receptor complexes, where chickens express over 100 receptors, a significantly expanded repertoire compared to mammals.^53^ Furthermore, chickens encode 10 TLRs of which five are human orthologs. For instance, the chicken TLR5 does not have a mammalian counterpart, and is specialized in the recognition of unique components of gram-positive and gram-negative bacteria, including *Salmonella.*^50^^,^^209^ Interestingly, chickens encode two IL-8 homologs, CXCLi1 and CXCLi2, which play pivotal roles in the immune response to *Salmonella* infection.^210^^,^^211^

At the time of hatching, the intestinal immune system is still underdeveloped. Significant developmental changes take place during the first 5 days after hatching, with key structures such as PPs and cecal tonsils developing only at 3–4 weeks post-hatch. As a result, young chicks show high susceptibility towards gastrointestinal infections.^54^^,^^212^

#### Gastrointestinal tract

The GIT of chicken is adapted to efficiently store and digest unmasticated food.^213^ A specific feature of the chicken GIT is the crop. This organ connects the esophagus with the stomach and functions as a specialized storage sac that temporarily holds food and initiates the fermentation process.^55^ The stomach itself is divided into two sections: the proventriculus, which is responsible for enzymatic digestion, and the gizzard, which mainly performs the muscular grinding of food.^214^ Another notable feature of the avian intestinal tract includes the presence of two ceca, which serve as the primary site for fermentation.^56^^,^^57^ This contrasts with humans, where fermentation predominantly occurs in the colon. In chicken, the colon is relatively short and plays a minor role. As zebrafish, chicken excrete both feces and urine together through the cloaca.^215^

The microbiome composition of chicken shares similarities with other warm-blooded animals at the phylum level, with Firmicutes and Bacteroidetes predominating. However, differences appear at the genus and species levels, particularly when comparing different segments of the GIT and how colonization changes with age.^58-60^

A key factor contributing to the distinct microbial colonization patterns in chicks compared to human infants is the mode of birth. In chickens, the initial microbiome is heavily influenced by the laying environment and the egg shell, whereas in humans, the early microbiome is shaped primarily by the maternal microbiome and breastfeeding.^216^^,^^217^ At hatch, young chickens are primarily colonized by Proteobacteria, particularly of the family *Enterobacteriaceae*. In humans, the first stable colonizers are dominated by facultative anaerobes, with *Enterobacteriaceae* also being present. As chicken mature, the proportion of Firmicutes (especially Clostridia) increases, followed by an increase in Bacteroidetes. In contrast, breastfeeding in human infants promotes colonization by *Bifidobacterium*, a genus within the phylum Actinomycetota. Eventually, *Prevotella* (Bacteroidetes) and Clostridia (Firmicutes) also become more dominant in humans.^218^

The composition and complexity of the chicken microbiome vary across the GIT, with microbial diversity and abundance increasing from the crop to the cecum. Unlike in humans, the microbial composition in the chicken colon is less diverse and more variable, resembling that of the ileum or cecum depending on the timing of sampling.^59^

#### *Salmonella* infection

Poultry are natural hosts to *Salmonella* serovars. The outcome of infection depends on the serovar, the age of the chicken, the inoculum dose, and the site of infection. For instance, *Salmonella enterica* serovars Pullorum and Gallinarum are host-specific serovars, causing pullorum disease and fowl typhoid, respectively.^219^^,^^220^ Both are septicemic diseases, with pullorum disease primarily causing acute infections in young birds, while fowl typhoid infections predominantly results in chronic or acute infections in adults.^221-223^ In addition to host-adapted serovars, chickens can also be infected by non-host-specific serovars such as Typhimurium and Enteritidis, resulting in paratyphoid infections.^224-226^ These paratyphoid infections in chicken cause NTS-like infections, characterized by diarrhea, inappetence, and dehydration. They should not be mistaken for paratyphoid fever in humans, which refers to the systemic disease caused by serovars Paratyphi A and C.^227^^,^^228^ Adult birds are often inherently resistant to these serovars, as further explained below, and therefore asymptomatically carry and shed the pathogen. In contrast, young birds commonly develop clinical disease characterized by diarrhea and mortality in severe cases. Given their higher susceptibility, lower inoculum doses are required to infect young chicks, and these birds tend to shed the pathogen intermittently for longer periods compared to adults.^224^^,^^229^ Notably, certain inbred chicken lines are either highly susceptible or resistance towards *Salmonella*, depending on certain genes and loci including chicken *TLR4, slc11a1* and *sal1.* Chicken TLR4, as human TLR4, recognizes LPS and thereby induces an immune response against *Salmonella.*^230^ *slc11a1* encodes a membrane-bound ion transporter localized to the phagosomal membrane and is important to control intraphagosomal replication.^231^ Lastly, the *sal1* locus encompasses genes such as *Siva* and *AKT1*, which regulate apoptosis of host cells.^232^ On top of that, differences in intestinal flow rates of major histocompatibility complexes, and heterophil circulation have been associated with *Salmonella* susceptibility and resistance.^225^

Once internalized, NTS mainly colonizes the cecum, followed by the large intestine and ileum.^61^ Colonization typically induces cecal and intestinal inflammation, characterized by the upregulation of pro-inflammatory cytokines such as IL-1β, IL-8, IL-17, and IL-22, and by heterophil infiltration. Following heterophil recruitment, macrophages and T cells are mobilized to the cecal and intestinal lamina propria.^63^^,^^64^ The inflammatory environment also drives shifts in the gut microbiome, reducing *α*-diversity and expanding the presence of specific taxa, such as members of the *Enterobacteriaceae* family.^233^ At later time points, *Salmonella* also invades and induces inflammatory responses in the liver. Inflammation typically persists for up to two weeks, after which cytokine levels return to baseline, and IgA and IgG mediate protective immunity.^62^

A wide range of studies indicates that intestinal colonization and internal organ invasion in chickens are dependent on SPI-1 and SPI-2.^65-67^ However, conflicting results exist, with some studies reporting that SPI-1- and SPI-2-deficient strains retain their colonization ability over long time periods.^67-69^ These discrepancies are likely due to differences in experimental set-ups, such as the specific *Salmonella* serovars used, the age of the chickens at the time of infection, and the duration of the study. In contrast, there is broader consensus on the role of flagella and LPS, both of which have been shown to play a significant role in invasion and colonization.^65^^,^^234-236^ The virulence factor AvrA has in turn been associated with persistent *Salmonella* infection by actively suppressing the host inflammatory response.^237^

Given the high susceptibility of young chickens to *Salmonella*, they serve as an excellent model for studying early host‒pathogen interactions, immune responses, and age-related resistance mechanisms. The availability of well-defined and specialized inbred lines further enhances their suitability for these analyses.^225^ Additionally, the growing repertoire of transgenic and gene-edited chicken models continues to expand, providing new opportunities for targeted genetic research.^238^^,^^239^

While chickens are larger and have a longer generation time than mice, they remain relatively small and their reproductive cycle is still considerably shorter than that of larger vertebrate models such as pigs and cattle. The chicken model is primarily valuable for investigating *Salmonella* transmission and elucidating the causes of foodborne outbreaks, rather than for translating results to human infections.

### Murine model

The murine model is the most commonly used vertebrate system to study NTS infections. It combines a complex immune response and gastrointestinal system with advantages such as relatively small size, ease of genetic manipulation, and manageable housing requirements.

#### Immunity

Mice mimic humans remarkably well. In terms of immunology, the overall anatomical features and immunological components are similar, though specific functions and pathways may differ.^70^^,^^71^ Mice have, for instance, a higher lymphocyte to neutrophil ratio in the blood, and have macrophages expressing nitric oxide, whereas this function is not well described for human macrophages.^240^^,^^241^

Interestingly, mice have Paneth cells instead of neutrophils expressing defensins.^242^^,^^243^ These Paneth cells are abundantly present in the cecum of mice, while they appear in only smaller amounts in the cecum and proximal colon in humans. Conversely, goblet cells are widely present in the intestinal crypts of the cecum to rectum in humans, whereas they are only abundant in the intestinal crypts of the proximal colon and to a lesser extent of the distal colon and rectum in mice.^73^^,^^243^^,^^244^ Furthermore, mice have relatively more PPs distributed throughout the small intestine, in comparison to humans, where PPs are more common in the ileum.^242^^,^^245^^,^^246^

There are also deviations in leukocyte transit times and cytokine/chemokine signaling.^70^^,^^73^^,^^242^ Examples of the latter include regulatory T cell induction and IL-17 signaling. In humans, IL-17 production induces IL-8. However, mice do not encode a homolog of IL-8 and have a lower number of neutrophils, suggesting a different outcome upon IL-17 signaling.^242^^,^^247-249^ Next, differences in B and T cell maturation, regulation, and population exist. For instance, mice, but not humans, produce B cells that express TLR4, which allows them to respond LPS in the gut in a T cell-independent manner.^242^^,^^250^

Overall, mice and humans mostly achieve similar outcomes upon immune simulation, though the underlying mechanisms may differ.

#### Gastrointestinal tract

Similar to intestinal immunity, the intestinal tract of mice exhibits key physiological similarities to those of humans in terms of anatomy and functionality. Still, due to their granivorous nature versus the omnivorous diet of humans, differences are present.^72^^,^^73^ For example, the mouse’s stomach consists of both a non-glandular and a glandular part, while humans only possess the latter. The non-glandular stomach of mice primarily functions as a storage sac, and is typically colonized by *Lactobacillus* spp due to its mild acidic pH (3-4).^251^^,^^252^ In contrast, the human stomach, with a much lower pH (~1), merely supports acid-adapted genera, such as streptococci.^253^

Next, mice have a longer small intestinal tract relative to their body weight, with a smooth outer mucosal layer that lacks the circular folds characteristic of the human small intestine.^136^ The absence of these folds in mice reduces the colonization potential of mucus-associated bacteria, which are more prominent in humans.^72^ The arrangement of the large intestine varies as well. On the one hand, the cecum is relatively large in mice, functioning as a microbial fermentation vessel, whereas it is rather small and of minor importance in humans. On the other hand, the colon is relatively large and sub-compartmentalized in humans, while it appears smaller and smoother in mice.^137^ Additionally, the human colonic mucus grows faster and maintains thicker layers with a well-defined submucosal layer, which is less defined in the murine colon.^254^ These anatomical and functional differences are likely to shape the microbial composition and density along the GIT.

Not only anatomical differences, but also the metabolic rate and retention time of foods can give rise to microbial compositional differences. Given that mice have higher energy demands, the retention time of food is shorter, and the generation interval of microbiota needs to be higher. ^72^ To maintain a stable gut microbiome, mice developed a mucus trap in the colon, which transports mucus and bacteria back to the cecum, and perform coprophagy.^255^

While the gut microbiota in mice and human are similar at the phyla level, with Bacteroidetes and Firmicutes being predominant, there are variations in their relative abundances at the genus level. For instance, *Prevotella*, *Faecalibacterium*, and ruminococci are more prevalent in humans, whereas lactobacilli, *Alistipes*, and *Turibacter* are more common in mice. Notably, mice harbor segmented filamentous bacteria in the terminal ileum, which have been linked to innate immune maturation and protection against colonization by *Salmonella.*^74-76^ In humans, these bacteria have been observed only in infants.^256^ Overall, great diversity exists along the murine GIT, with the most diverse and dense population being present in the cecum and colon.^77^ Note that comparative analysis between the murine and human microbiomes is not straightforward owing to the wide variety of sequencing platforms, primers, and analysis pipelines used in distinct studies. Additionally, the microbial composition of mice can vary significantly depending on the utilized strain, diet, and housing conditions, making it difficult to compare studies directly.^257-259^

#### *Salmonella* infection

Despite the above-mentioned distinctions, murine models remain the premier vertebrate model systems for studying *Salmonella* infections. Notably, various mice strains show varying susceptibility to *Salmonella* administration. For instance, CBA/J and 129/Sv mice appear genetically resistant towards *S.* Typhimurium, whereas C57BL/6 and BALB/c strains are more sensitive to infection. This discrepancy is primarily due to a mutation in the *Slc11a1* gene.^78-81^ This gene encodes a natural resistance-associated macrophage protein 1 (Nramp1), which functions as a key iron transporter in macrophages.^80^^,^^260^ Functional Nramp1 limits *Salmonella* survival in macrophages, controlling replication in organs such as mesenteric lymph nodes.^261^ In addition to Nramp1, a broad range of genetic differences influencing pro-inflammatory signaling pathways, T cell function, antibody responses, and natural killer cell activity further determine strain-specific susceptibility.^262-265^ Overall, NTS infection in sensitive mice strains predominantly results in system infection,^266^ while in humans, they typically cause localized gastrointestinal infections.^267-269^ As such, the mouse model was initially used to study typhoidal *Salmonella* infections.

To better simulate NTS infection in mice, Bohnhoff et al. (1954) developed a streptomycin-pretreated murine colitis model, which was later redefined and popularized by Barthel et al. (2003).^84^^,^^87^ The streptomycin pretreatment disrupts the microbiome, lowering colonization resistance and thus aiding *Salmonella* to establish infection in the GIT. Here, oral administration of *S.* Typhimurium with an inoculum density of approximately 10^8^ CFU results in colitis, characterized by edema in the submucosa and lamina propria, rapid regeneration of intestinal epithelial cells, loss of goblet cells, pronounced polymorphonuclear neutrophil infiltration, and high inflammation of the intestinal lumen.^84^^,^^85^ Inflammation primarily occurs in the cecum and colon, with studies showing the expression of pro-inflammatory cytokine IFN-*γ*, and inflammasome-dependent activation of IL-22 and IL-17 in the cecal mucosa. T-cell-mediated-induction of IL-23 further amplifies this response.^82^^,^^83^ The inflammatory signaling subsequently results in the production of downstream products such as inducible nitric oxide synthase and lipocalin-2.^270^ Compared to humans, luminal fluid secretion remains relatively mild. While the systemic spread of *Salmonella* is substantially lower than in streptomycin-untreated mice, dissemination to internal organs such as the spleen and liver still occurs.^84^

Multiple *Salmonella* virulence factors contribute to disease progression in the murine colitis model. Flagella, for instance, enhance mucus penetration, SPI-1 effectors drive cecal inflammation and colonization, and SPI-2 effectors promote intracellular survival.^86^ Still, the relative contribution of these virulence factors appears to vary depending on the serovar examined.

Several variations of the streptomycin-pretreated murine model, including adaptations in inoculum density and administration route, have been validated,^268^ and various alternative models have been proposed. One substitute involves using germ-free mice instead of antibiotic-treated ones.^90^ In this set-up, *Salmonella*-induced colitis displays similar infection kinetics, depending on analogous virulence factors, but the pathology is more severe.^271^ Alternatively, gnotobiotic mice, which are born germ-free and are subsequently colonized with a defined microbial community, can be used to dissect specific interactions between *Salmonella* and individual microbiome members.^88^^,^^89^ Here, humanized mice, colonized with human-derived microorganisms, allow investigation of human host–pathogen–microbiome interactions. However, because host–microbiota relationships arise from long-term co-evolution, these reconstructed systems cannot fully recapitulate the complexity of human-specific interactions, and thus require careful interpretation. Another model involves the use of resistant CBA/J or 129/Sv mouse strains without streptomycin pretreatment. In this case, *Salmonella* infection initially causes acute colitis, resembling that in C57BL/6 mice, but progresses to chronic intestinal inflammation between days 7 and 43 post-infection.^81^^,^^272^ As such, this model allows the study of chronic, long-term *Salmonella* infection. Furthermore, given the lack of antibiotic-pretreatment, this model is often used to study the effect of *Salmonella* infection on the microbiota.

Mice allow elegant investigation of distinct aspects of the infection process. First, the interaction between *Salmonella* and the immune system can be explored using either specific immune-deficient strains, such as the NOD-SCID-gamma strain, or through the routine administration of immunosuppressive agents.^273-276^ Second, interactions with the microbiome can be examined using germ-free, specific-pathogen-free, or microbiota-transplanted mice.^89^^,^^259^^,^^277^^,^^278^ Third, spatial dynamics can be investigated by changing the inoculation route, administering *Salmonella* either orally, intraperitoneally, or intragastrically. Finally, advancements in microscopy, such as near-infrared microscopy for *in vivo* imaging, and fluorescence in situ hybridization (FISH) imaging for detailed structural analysis, enhance the ability to study infection dynamics at high resolutions.^279-282^ Mice are thus highly valuable in gaining mechanistic insights into the infection process.

### Bovine model

Analogous to chicken, cattle represent a natural reservoir for *Salmonella,* with *S.* Typhimurium and Dublin being the predominant serovars. Given that *S.* Typhimurium-induced gastroenteritis establishes similarly in cattle and in human, cattle are a commonly used experiment model systems to study NTS.^96^

#### Immunity

The immune system of cattle highly resembles that of humans, sharing key innate and adaptive cell types, signaling cascades, and pathogen recognition pathways.^105^ Delving deeper into the different components, a few minor differences exist. For example, bovine neutrophils express both NOD1 and NOD2, whereas human neutrophils express only the latter. Furthermore, while humans produce both *α*-defensins and *β*-defensins, cattle exclusively express *β*-defensins.^283^ Another distinction is the presence of two types of PPs in cattle, including discrete patches in the jejunum and a continuous patch in the terminal ileum.^284^ Cattle also possess three distinct types of IgG and two types of IgM classes, with IgG1 being the dominant immunoglobulin in mucosal secretions.^285^^,^^286^ This contrasts with most other animals, where IgA primarily provides mucosal protection.^287^ Additionally, compared to other mammals, young calves possess a significantly higher proportion of γδ T cells, likely to compensate for their immature neutrophils and macrophages during early life.^288^ However, all above-mentioned distinctions have a rather minor impact on the progression of bacterial infections.

#### Gastrointestinal tract

The GIT of cattle is specifically adapted to process fibrous plant material, and therefore substantially differs from that of omnivores such as human and pigs. First, cattle have a four-compartment stomach, comprising the rumen, reticulum, omasum, and abomasum.^92^ Here, the rumen and reticulum function as a large fermentation sac, hosting a rich microbiome which is specialized in breaking down plant material. This fermentation process typically results in volatile fatty acid production, which serve as an energy source for the host.^289^^,^^290^ Eventually, the liquid portion remaining after fermentation moves further to the omasum, which focusses on the absorption of nutrients.^291^ The abomasum in turn serves as a true stomach, containing hydrogen chloride, pepsin and maintaining a low pH.^92^^,^^292^

Following transit through the four stomachs, the digesta travels through the small and large intestines for nutrient absorption and additional fermentation, respectively. Compared to humans, the small intestine, cecum, and large intestine are larger in cattle and more specialized in processing microbial proteins and fermenting fiber.^92^ The ascending colon is subdivided into a proximal loop, spiral colon, and distal loop, and an appendix is lacking.^293^

Firmicutes and Bacteroidetes are the dominant phyla in the bovine microbiome.^93^^,^^94^ The reticulorumen hosts the most diverse and densely populated microbial community, which is primarily composed of anaerobic and methanogenic genera. Here, the exact composition varies depending on the location. For example, particle-associated bacteria are dominated by *Ruminococcus* spp. and biofilm-forming genera, while the liquid fraction is primarily composed of amylolytic and proteolytic bacteria from the phylum Bacteroidetes. In contrast, tissue- and epimural-associated microbiota are enriched in aerotolerant Proteobacteria, which play a crucial role in reducing oxygen diffusion from the bloodstream.^93^^,^^94^ Additionally, short-chain fatty acid producers such as *Alistipes*, Bacteroides, and Clostridia are frequently detected in bovine fecal samples, reflecting the efficiency of fiber fermentation.^94^ The composition of the bovine microbiome can be further influenced by various factors, including diet, housing conditions, and exposure to infectious agents, such as *Salmonella.*^294^

#### *Salmonella* infection

Cattle form a natural reservoir of *Salmonella* serovars, possibly transmitting the disease to humans through the ingestion of contaminated food. Depending on the *Salmonella* serovar, the inoculum dose, and the age of the animal, the pathogen may cause clinical disease. The two most common serovars associated with cattle are *S.* Dublin and *S.* Typhimurium.^96^^,^^295-297^ The former is highly invasive in young calves, causing, beside diarrhea, meningoencephalitis, polyarthritis, osteomyelitis or pneumonia. In humans, *S.* Dublin infection typically results in bacteremia.^298^^,^^299^ In contrast, infection with *S.* Typhimurium leads to enteric disease, characterized by diarrhea, anorexia, and fever, in both cattle and humans.^97^^,^^103^

*S.* Typhimurium predominantly colonizes the ileum in cattle, proliferating within the lumen and invading the epithelial and M cells.^95-97^ Similar as in humans, tissue invasion induces inflammation, characterized by the expression of several chemokines, including IL-8 and growth-related oncogene *α* (GRO-*α*), and cytokines, such as IL-1β.^100^^,^^300^^,^^301^ The chemo- and cytokine signaling recruits neutrophils to the site of infection, which in turn leads to necrosis of the ileal mucosa and ultimately results in severe fluid loss.^96^^,^^98^^,^^99^ SPI-1 and SPI-2 effectors are thought to be important for enteric and systemic infection.^100^^,^^101^

Since *S.* Typhimurium-induced gastroenteritis presents similarly in both cattle and humans, cattle are frequently used as a model organism to study NTS. This research typically involves infection in calves or the use of the ileal-loop model.^8^ During infection in calves, young animals (≤ 2 months old) are orally challenged with *Salmonella* using an inoculum size of approximately 10^8^. Here, animals rapidly develop clinical features with diarrhea within 48 hours.^103^ Using the ileal-loop model, calves are terminally anesthetized, a laparotomy is performed, and up to 10 cm loops are prepared within the distal ileum, which can be inoculated with *Salmonella.*^102^ The possibility to create multiple loops allows the inclusion of positive and negative controls within one animal, thereby reducing the number of animals needed. With infection kinetics resembling those of orally-infected calves, this model is particularly suitable for studying early timepoints of infection.^302^ Initially, the model was limited to time points within 12 hours post-infection, but restoring small intestinal patency allowed for extended observations.^303^

In conclusion, the bovine model effectively replicates human infection while also providing ecological relevance, as cattle serve as a natural reservoir for *Salmonella*. However, this model is not always straightforward to implement owing to high maintenance costs, ethical considerations, and the logistical challenges associated with working with large animals. Additionally, fewer genetic and immunological methods are available for cattle compared to other models.

### Pig model

Domestic pigs resemble humans closely in terms of anatomy, genetics, and physiology.^104^ Besides, pigs are natural hosts to *Salmonella* and therefore represent ideal model organisms to study NTS infection.

#### Immunity

The porcine immune system is well-characterized and exhibits significant anatomical, structural, and functional similarities to the human immune system.^104^^,^^105^ On the anatomic level, pigs, for instance, also possess palatine and pharyngeal tonsils and retain a spleen tightly attached to the stomach. Furthermore, on the functional level, pigs utilize the chemoattractant IL-8,^304^^,^^305^ produce macrophages without nitric oxide production,^306^ and maintain a high proportion of neutrophilic granulocytes in peripheral blood,^307^ similar to humans.

Some minor differences in the immune organization still exist. For instance, similar to cattle, pigs encode both jejunal and ileal PPs.^308^^,^^309^ Other examples include the co-expression of CD4 and CD8 by T cells outside the thymus,^310^ the absence of *α*-defensins,^311^ and the lack of NOS2 induction in response to LPS.^306^ However, as with cattle, these discrepancies are of minor relevance when comparing the course of NTS infection between pigs and humans.

#### Gastrointestinal tract

The intestinal tracts of pigs and humans share striking similarities owing to their omnivorous nature. For instance, both species contain a single glandular stomach, composed of cardiac, gastric, and pyloric mucosa.^106-108^ However, the pig’s stomach comprises significantly more cardiac mucosa, which shapes a pseudo-diverticulum that functions as a storage and digestive bag. This pseudo-diverticulum forms a unique niche for commensals that is not found in humans.^108^

The human and porcine small intestines are comparable in terms of relative length, villi structure, and transit time.^312^ Still, the arrangement of the small and large intestines differs slightly, with pigs for instance having a relatively longer duodenum and colon. Additionally, pigs rely on substantial cecal fermentation, possessing a relatively large cecum, whereas humans primarily engage in colonic fermentation.^106-108^ Other differences include the absence of an appendix in pigs, and the presence of a spiral-fashioned colon.

Similar to other warm-blooded animals, the porcine microbiome at the phylum level is predominantly composed of Bacteroidetes and Firmicutes.^109-112^ Several studies have attempted to characterize the microbiome on the genus level, but the composition seems to depend substantially on the pig strain, age of the pigs, housing and feeding conditions, and utilized DNA extraction kits.^108^^,^^110-113^^,^^313^^,^^314^ As in most animals, the microbial community transitions throughout the different growth stages, with increasing *α*-diversity and complexity over time. At birth, piglets are initially colonized by facultative anaerobes, followed by successive waves of bacterial colonization. In finishing pigs, the gut microbiota is dominated by *Prevotella* and Clostridia, whereas in humans, the final colonization wave primarily results in a microbiome dominated by Clostridia and Bacteroides.^111^^,^^315^ In both pigs and humans, microbial composition and density vary along the GIT, with greater microbial richness and density observed towards the colon.^113^

#### *Salmonella* infection

NTS infections in pigs are well-described and are typically established through the fecal-oral route. The outcome of infection depends on the inoculation dose, where pigs may either develop self-limiting diarrhea or enter an asymptomatic carrier state.^316^^,^^317^ In *Salmonella* research, pigs are studied either via oral infection of piglets or using porcine ileal loop models, which operate on the same basic principles as bovine loops.^122^^,^^123^ To model gastroenteritis in piglets similar to that in humans, experimental infections are typically performed with a minimum of 10⁸ CFU. Under these conditions, the pathogen becomes detectable in the feces within 24 hours. Interestingly, three groups can be distinguished depending on the shedding kinetics: low shedders, intermediate shedders, and high shedders. These groups do not only differ in the degree of *Salmonella* shedding, but also in the extent of colonization and inflammation, with the high shedders showing the most pronounced effects.^124^

In the high shedders, clinical signs such as fever, diarrhea, and lethargy readily develop, peaking at 2 days post-infection.^124^^,^^313^^,^^317^^,^^318^ After rapidly colonizing the palatine tonsils, *Salmonella* travels to the mandibular lymph nodes and the GIT. Here, colonization mainly occurs in the ileum, followed by the cecum and colon, with the jejunum being less affected, likely due to its higher concentration of bile salts.^114-116^ Intestinal inflammation is mediated by TLR-4, MyD88, and NF-κβ signaling, and is characterized by the upregulation of pro-inflammatory cytokines, including IL-1β, IL-6, TNF-*α*, and IFN-*γ*, and the chemokine IL-8. IL-8 typically recruits neutrophils to the site of infection.^117^ The resulting inflammatory environment is associated with reduced gut microbial *α*-diversity and the expansion of facultative anaerobic, microaerophilic, and aerotolerant taxa. Usually, the inflammatory response begins to resolve, and severe diarrhea subsides after 6 days.^114^^,^^319^

Intestinal colonization, invasion, and inflammation appear to be SPI-1 dependent.^118-120^ Contrarily, colonization of the palatine tonsils did not rely on SPI-1 effectors, with SPI-1 mutants showing equally high colonization rates and cytokine signaling suppression as wild-type *Salmonella.*^120^^,^^320^ Notably, the pathogen remains extracellularly in the tonsils, and thus presumably does not rely on SPI-1-dependent invasion for colonization of this tissue. SPI-2 does not seem important for intestinal infection, but contributes to systemic colonization.^121^

After clinical recovery, a small proportion of pigs can become carriers, intermittently shedding *Salmonella* for up to 5 months. This subclinical disease is typically associated with colonization of the ileocolic lymph nodes, the palatine tonsils, and, to a lesser extent, the GIT.^313^^,^^316^^,^^319^ Due to intermittent shedding, *Salmonella* cannot always be detected in the feces effectively. Seroprevalence based on anti-*Salmonella* IgG might be a better way to detect chronic carriers.^321^^,^^322^

Pig models provide valuable information for studying both acute and chronic NTS infections. Similar to mice, immune-deficient pigs enable the study of the interaction of *Salmonella* with the immune system.^323^^,^^324^ Additionally, pigs have gained significant attention in microbiome research, with the advancement of specialized models such as germ-free, gnotobiotic, and human-microbiota-associated pigs.^108^^,^^325^^,^^326^ As with mice, non-invasive *in vivo* imaging techniques and FISH imaging on fixed tissues can be employed, offering detailed insights into infection dynamics.^327^^,^^328^ While pigs represent the clinically most relevant model organisms, their implementation can pose challenges in terms of housing, handling, and costs, and is therefore not always a straightforward option.

## Conclusion

*In vivo* models play a crucial role in uncovering mechanistic insights into pathogen‒host interactions, offering valuable information that cannot be obtained from *in vitro* systems alone. Based on *in vitro* studies and experiments in the *vivo* models depicted above, a general mechanistic model for NTS infection has been established. In this model, the NTS colonizes and invades epithelial cells through the coordinated action of the effector proteins encoded on SPI-1. This invasion induces epithelial signaling cascades that promote cytoskeletal rearrangement and bacterial internalization, while triggering a robust inflammatory response.^329-331^ The ensuing inflammation would paradoxically enhance luminal *Salmonella* expansion by altering the intestinal microenvironment in favor of the pathogen.^85^^,^^332^^,^^333^ The epithelial-internalized bacteria would in turn reside within a modified phagosomal compartment known as the *Salmonella*-containing vacuole (SCV).^334^^,^^335^ The maturation and maintenance of this SCV largely relies on effector proteins encoded on SPI-2.^336^ In addition to epithelial invasion, *Salmonella* could be translocated via the M cells overlying PPs and survive within macrophages, stimulating systemic spread.^337^ Several aspects of this generalized framework can vary depending on the host or experimental system. For instance, differences can arise in the primary site of colonization, the characteristics and magnitude of the inflammatory response, and the relative contribution of individual virulence factors. Whether the described patterns fully translate to human infection remains to be determined.

A wide set of animal models exists, ranging from simple, cheap, high-throughput invertebrate models to more complex and costly but physiologically relevant vertebrate models. Each model has its own advantages and limitations. Invertebrate models such as *C. elegans* can, for instance, be useful for high-throughput screening and imaging, providing preliminary insights into host‒pathogen interactions. However, these models contribute little to the understanding of *Salmonella*’s ecological role and are physiologically rather distant from humans. The vertebrate zebrafish and murine models are physiologically closer to humans, but their implementation remains highly artificial. As such, these models similarly do not provide ecological insights.

In contrast, chickens serve as important natural reservoirs of NTS, making them highly relevant for studying transmission and persistence in the environment. However, findings from chicken models cannot be directly extrapolated to human infections because of key physiological differences. Cattle and pigs, on the contrary, offer the closest resemblance to human infection, both in terms of gastrointestinal physiology and immune response, while also serving as natural reservoirs of *Salmonella*. Despite their relevance, these large animal models present practical challenges, particularly in terms of handling, housing, and cost. In conclusion, each *in vivo* model has its own strengths and limitations, and no single model can answer all questions. Instead, a complementary approach, leveraging insights from multiple models, is essential for gaining a comprehensive understanding of both infection dynamics and ecological relevance.
